# An Integrative Program to Reduce Stigma in Primary Healthcare Workers Toward People With Diagnosis of Severe Mental Disorders: A Protocol for a Randomized Controlled Trial

**DOI:** 10.3389/fpsyt.2019.00110

**Published:** 2019-03-07

**Authors:** Pamela Grandón, Sandra Saldivia, Pamela Vaccari, Raul Ramirez-Vielma, Víctor Victoriano, Carlos Zambrano, Camila Ortiz, Felix Cova

**Affiliations:** ^1^Department of Psychology, University of Concepción, Concepción, Chile; ^2^Department of Psychiatry and Mental Health, University of Concepción, Concepción, Chile; ^3^Group of former Mental Health Users, Concepción, Chile

**Keywords:** stigma, severe mental disorders, clinical trial, primary care, healthcare workers

## Abstract

**Background:** People with severe mental disorders (SMDs) have higher disease and death rates than the general population. Stigma (negative attitudes and perceptions) contributes to limited access to health services and a lower quality of medical assistance in this population, and it is manifested as negative attitudes, social distance, and discrimination toward this social group. For these reasons, healthcare workers are a priority group for anti-stigma interventions. This study aims to assess the effectiveness of a program specifically designed to decrease negative attitudes and social distance and increase inclusive behaviors in healthcare workers toward people with SMD.

**Methods:** The study will be a randomized clinical trial. A minimum of 210 healthcare workers from 11 primary care centers in the province of Concepción, Chile, will be randomly chosen to receive the program or be part of the control group. There will be a pre-, post-, and 4-months evaluation of social distance, attitudes, and behaviors of participants toward people with SMD using standardized scales such as the social distance scale, which is a scale of clinician attitude toward mental illness adapted from attitudes of clinicians toward mental illness, and self-reports. The intervention program will consist of education strategies, direct, and indirect contact with people diagnosed with SMD, and skill development. There will be six face-to-face sessions directly with the participants and two additional sessions with the directors of each healthcare center. The program will involve a facilitator who will be a healthcare professional and a co-facilitator who will be a person diagnosed with SMD.

**Discussion:** This study will evaluate an intervention program especially designed to reduce stigma in healthcare workers toward people with SMD, a topic on which there is little background information, particularly in low- and middle-income countries. It is important to have interventions with proven effectiveness for this purpose to ensure equity in healthcare services.

**Trial Registration:** This study was registered under ISRCTN.com (ISRCTN46464036).

## Introduction

Several studies have shown that people with severe mental disorder (SMD) have higher morbidity, lower life expectancy, and higher mortality rates than the general population (1, 2). There is no consensus on the definition for SMD and on the specific disorders it comprises. However, in general, the criteria proposed by the National Institute of Mental Health (NIMH) in 1987, which continue to be used, are as follows: diagnosis of psychosis, duration of more than 2 years, and marked interference in daily functioning (3–5). Schizophrenia is often considered the prototype of SMD (6).

Studies have shown that people with SMD have a higher prevalence of hepatitis, osteoporosis, obstetric complications, cardiovascular diseases, obesity, diabetes, dental problems, and other chronic diseases compared to the rest of the population (7, 8). Furthermore, these results have been found in countries with different cultural backgrounds, including those in Latin America, such as Brazil (9–11). It has also been shown that people with SMD are less likely to receive adequate health care (12). Indeed, there exists an inequality in health care for this population compared to the general public, both in hospital care and primary healthcare service (PHS) (13). Hence, there is an interest in developing strategies to ensure equity in health care for people with SMD (14–16). Inequity in health care is a result of multiple factors, but the more unresolved one is stigma toward people with a diagnosis of SMD (17, 18). Stigma is currently understood as a relational process that includes cognitive, affective, and behavioral components. It refers to a social process of labeling, loss of status, and discrimination toward a person who has an attribute that is considered negative by their community (19). Self-stigma is one of the main consequences of stigma and is characterized by the loss of self-esteem and self-efficacy due to the internalization of public stigma. Living in a society that ascribes negative characteristics to people with SMD can lead them to internalize these ideas and think that they are less valuable because they have a psychiatric disorder (20). Moreover, self-stigma has other harmful effects because some people will not seek professional help or follow treatment for fear of being identified as “mentally ill” (21). Furthermore, self-stigma produces feelings of shame and anxiety in people with SMD that can increase their stress and affect their risk of relapse (22).

In health care, several studies indicated that healthcare personnel have prejudices, negative attitudes, and discriminatory behavior toward people with SMD (16, 23–25). For instance, a recent study comparing professionals at both PHS and secondary healthcare centers found that physicians and nurses at PHS had more negative attitudes toward people with SMD than their colleagues at secondary healthcare centers (26). Although stigma is similar in different professional groups such as nurses (27, 28), physicians (29), pharmacists (30), and psychologists (31), there seems to be some differences between them. For example, professionals closest to the area of social sciences, such as psychologists, would have less stigma than professionals in the area of healthcare, such as physicians and nurses (32). Despite the few studies on this topic in Chile, the results are in agreement with other published studies demonstrating that there are prejudices and negative attitudes toward people who have SMD (33, 34). It has been shown that stigma affects the inequity of health care in several ways. For instance, there is abundant evidence describing the effect of barriers to access healthcare services that makes it difficult for the affected people to request help (35, 36). In Chile, the lack of knowledge about mental disorders and stigma are the main reasons Chileans do not seek treatment (37). On the other hand, healthcare personnel mistakenly attribute physical signs and symptoms to a mental disorder, which leads to sub-diagnosis and inadequate treatment of physical health problems (16). Finally, a negative attitude toward the patient is associated with a lower adherence to treatment. Therefore, healthcare personnel should be considered a priority group for anti-stigma interventions, especially those working in PHS (38).

The area of anti-stigma interventions is a relatively new field. One of the models used to reduce stigma is the social-cognitive model (39). In this model, erroneous social beliefs (i.e., stereotypes) produce a negative attitude (i.e., prejudice) that leads to discrimination. The prejudice is based on various settings and previous biases that tend to favor the perception of the ingroup over that of the outgroup. In other words, everything that seems strange or alien to one's own perception tends to be categorized in a negative way. Thus, the intervention is focused on modifying stereotypes and attitudes to influence discriminatory behavior (40). For example, stereotype perception is modified by providing information that disproves previous concepts, and prejudices are reshaped by favoring processes of re-categorization to develop new ideas and attitudes. These processes are carried out through social interaction with the affected outgroup (41, 42). However, studies in this area show that giving information by itself does not assure a behavioral change, but that modification of the emotional response (prejudice) is more effective at getting people to act differently (43). The emotional change is easier to achieve through direct interaction with stigmatized people, thus the subject or situation that is alien or foreign becomes familiar and close.

Two major types of anti-stigma campaigns have been developed: generalized and localized. The first corresponds to mass campaigns developed from different strategies aimed at the general public (44). Among the most outstanding initiative in this area is the program “Open the Doors” (World Psychiatric Association) (42). Despite the benefits of this type of campaign, several researchers maintain that it has important limitations, such as: (i) little knowledge of the population, (ii) biases in the evaluation, and (iii) low efficiency to internalize what has been learned. Hence, other types of more localized or workplace interventions have been proposed as preferable alternatives. These initiatives are aimed at selected groups such as employers, schoolchildren, and healthcare personnel, among others; they have the advantage of being more intensive, so that although they reach fewer people, more work is done with the participants. On the other hand, it has been demonstrated that information against attitudinal learning is only assimilated in the context of where it is taught; thus, it is better to acquire it in places where it will later be used (45). For example, a recent review by Hanisch et al. (46) indicated that anti-stigma interventions in the workplace are the most effective at reducing stigma in the work environment. Programs in the workplace have been fundamentally based on two major strategies or components: education and contact. Education provides information about mental disorders to modify social stereotypes. For instance, specific aspects of SMD are discussed such as causes, risk factors, symptoms, and treatments (47). This education is useful if the transmitted information is discussed and real cases are presented. It is also necessary to establish the types of beliefs to be modified according to the particular diagnosis and the characteristics of the intervention group. This type of intervention is mainly done through conferences and debates (48). Furthermore, several studies have shown that knowledge about the subject makes people less likely to stigmatize and discriminate against this population (43, 49). This strategy has been one of the most utilized with healthcare personnel, and similar to what happens in the general population, the results indicate that imparting information decreases negative attitudes toward people with SMD; however, the duration of the changes is limited (50).

The other major strategy, contact with affected people, is limited to interventions in which a person with a mental disorder tells their experience to a specific group, either directly or indirectly through audiovisual media (48, 51). The conditions under which contact occurs are important for success. For example, the interaction should be cooperative, perceiving similar status, and not competitive. Indeed, the largest meta-analysis performed on the subject, which included 515 studies, determined that the conditions of contact raised by Allport contributed to diminishing prejudice; however, even if all conditions are not met, contact has a positive effect on decreasing stigma (52). For contact to be effective, it is important that people do not have behaviors and attitudes that are stereotypical of the SMD, and that they have the support of the directors of the Center where the intervention is carried out. Contact seems to be the best strategy to reduce stigma against people with SMD. In fact, several studies indicate that subjects who interact with people who have SMD are less likely to have negative attitudes toward them (53, 54). In a study performed in Chile on the perception that the general public and healthcare workers have toward people with SMD, it was found that those who had greater contact with people with SMD had less authoritarian and restrictive attitudes (55). Indeed, studies performed with healthcare personnel demonstrate that using direct contact with people with SMD is a strategy that decreases negative attitudes and social distance and increases acceptance toward this social group (23, 56, 57). However, the main limitation to developing this strategy is that it requires that people affected by a mental disorder be willing to publicly disclose their condition and talk about it. Despite this difficulty, studies suggest that the active involvement of those affected in these interventions increases their self-esteem and empowerment, which are fundamental for their recovery (54).

Intervention programs tend to use both strategies in different formats. Although there is evidence that education and social contact reduce prejudice toward people with SMD, it is unclear under what conditions each strategy would be more effective (44). In recent years, some research has shown that healthcare personnel have more negative attitudes toward people with SMD when they feel that it is difficult to interact with them and they are unable to contribute to their treatment (58, 59), which leads to frustration and rejection. Thus, assessments of anti-stigma programs recommend including skills training (60–62). For example, teaching healthcare personnel how to welcome and resolve difficult situations with those who have SMD and their families is a central factor in reducing stigma.

Intervention programs have been evaluated mainly through tests that measure people's knowledge and attitudes toward SMD, where knowledge tests indicate the level of information acquired by people post-intervention (63). With regards to attitudes, multiple scales have been used, the most common one being the Community Attitudes to Mental Illness (CAMI) scale (63). Social distance measures are also used to assess the willingness of someone to interact with a specific person in a specific type of relationship (64). These scales evaluate behavioral intention, so they are in some way between the evaluation of attitudes and behaviors. Therefore, these are used as a proxy measure for behavioral change. This is important since studies have pointed out that in addition to considering attitudes, one must also evaluate the behavior modification of participants in an intervention. Indeed, a behavioral change toward people with SMD would ultimately improve their quality of life (47). However, the main limitation of studies on anti-stigma interventions is that they used a pre- and post-test design without a control group. It was estimated that < 30% of studies used a control group, so the interpretation of their results for the intervention cannot be considered statistically valid (41, 44). In the last decade, some researchers have suggested that for an intervention to be successful, it is necessary to perform a qualitative evaluation before and after the intervention and to actively involve people diagnosed with SMD (47). This type of evaluation makes it possible to understand the nature of the stereotypes and prejudices of the group that one is going to work with. This would allow the implementation of an intervention relevant to the beliefs of the target group (40).

Regarding the participation of service users, an approach called “Research based on community participation” has been promoted for several years (65). This encourages the participation of affected people in their health care throughout the research process, both in the design of the interventions as well as in the execution and evaluation. Several studies point out that anti-stigma interventions should be performed from this perspective so that the programs respond to the needs of those affected while at the same time favor their recovery and empowerment (50, 63). Therefore, it is fundamental to reduce negative attitudes and discrimination in PHS personnel toward this social group. However, there is little international research on anti-stigma interventions with healthcare personnel, especially in developing countries (66). This is a relevant fact because context has an important role in the effectiveness of an intervention; in other words, cultural differences in attitudes toward people with SMD are crucial for the design of the intervention (47).

The present study trial seeks to evaluate a mixed intervention strategy for the reduction in stigmatization associated with SMD, particularly in people diagnosed with schizophrenia. To accomplish this, an intervention program will be designed, implemented, and evaluated. This program combines methods/techniques that have been shown to provide the best results in reducing stigmatization. The program will implement education, contact, and skills development as core strategies. We will actively work with people who have received a diagnosis of SMD. The objective is to design, implement, and evaluate an intervention program to reduce negative attitudes and social distance, and increase inclusive behaviors of healthcare workers toward people with SMD. The program will be implemented with healthcare workers from primary care centers. In Chile, the majority of the population use primary healthcare centers for medical attention in the public health system. Each center provides service to a specific number of inhabitants located in the population sector, and is equipped with a multi-professional health team, technicians, and an administrative support staff. The intervention program will be developed for all healthcare workers except the administrativ staff.

The hypotheses for this study are as follows:

Compared to the control group, healthcare workers in the intervention group will have less negative attitudes and social distance toward people with SMD (schizophrenia) in the post-test evaluation and follow-up.
Healthcare workers in the intervention group will exhibit more inclusive behaviors toward people with SMD (schizophrenia) in the post-test evaluation and follow-up compared to the control group.

## Methods

### Design

**Figure 1:**
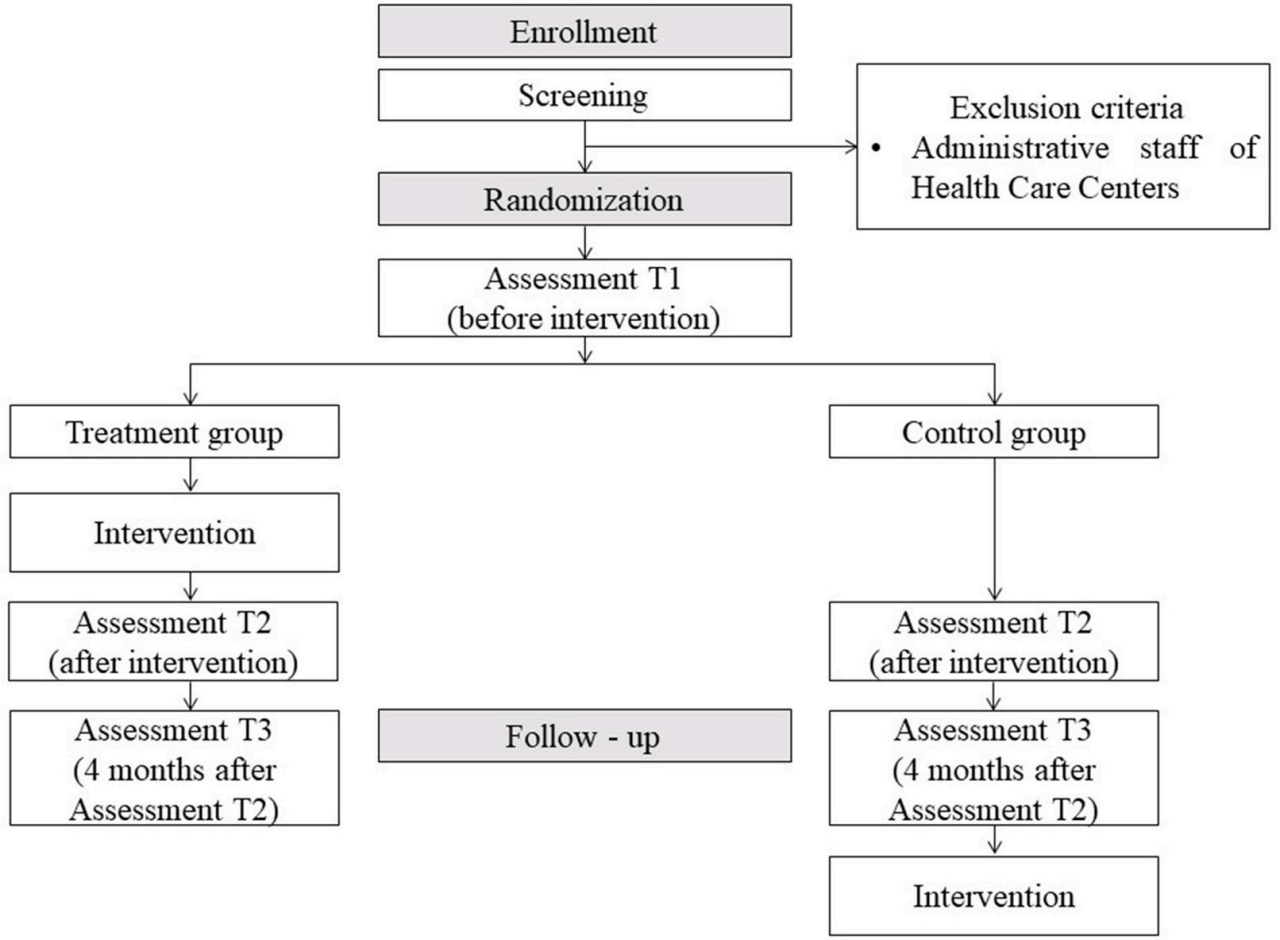
Study flowchart.

The study design is a multicenter randomized clinical trial in which several primary healthcare centers in the province of Concepcion, Chile will participate. Randomization will be at the individual level (Figure 1). The randomization will be performed by blocks (each healthcare center will be a block) and will be performed after identification of all the people who have agreed to participate in the study. Two stratifications will be made for each block. The first stratification will be by the type of professional involved and divided into three categories: physicians, health professionals (nurse, midwife, nutritionist, physical therapist, dentist, paramedic, pharmacist, podiatrist), and psychosocial professionals (psychologists and social workers). The second stratification will be by the work sector of each healthcare center. In each stratum, a similar number of people will be randomized for the experimental and control group using the software package “blockrand,” which creates randomizations for block random clinical trials.

### Intervention Program

The objective of the program is to reduce negative attitudes and social distance, and increase the inclusive behavior of healthcare workers toward people with SMD.

### Program Development

Focus groups were conducted with people diagnosed with SMD and primary healthcare workers to identify the views that both groups have about how stigma may be expressed in healthcare. This information was used to design the program. Once designed, a panel of experts composed of an academic specialist on the subject, a health professional from PHS, a patient from a PHS with a diagnosis of SMD, a person with SMD who participates in social organizations, an organizational psychologist who specializes in training programs, and a health professional from the secondary level were asked to review it and make suggestions. The program was subsequently implemented as a pilot trial at a healthcare center to refine it and make it more relevant. A manual was developed detailing the protocol for the intervention and explaining the specifics of each activity per session.

The intervention program was designed considering a research approach based on community participation and available background information on strategies that would be most effective. A unique aspect of this intervention is its “ecological” perspective that takes into consideration various structural levels and processes of the social environment and their effect on individual behaviors, thoughts, and emotions both during the formation and subsequent modification of the stigmatization process (67).

### Characteristics and Methods of the Program

The program considers the following levels of intervention:

#### Organizational/structural

The intervention involves aspects of the organizational culture and the structure of the healthcare system that contributes to and influence stigmatization processes (68), such as norms, values, and attitudes (47).

#### Intergroup

Here, the intervention evaluates two central processes: biases derived from group identities (69), and the increase in prejudice associated with contact between social groups having conditions of inequality (70).

#### Individual

At this level, the intervention intends to modify beliefs and attitudes using the social cognitive theory, which is a model based on associative-propositional assessment of attitudes, and the theory of cognitive dissonance (71, 72). In an implementation of the program, a group of participants were people who were diagnosed with a SMD but who had been discharged after their rehabilitation was finished. This organization of people is involved with groups and communities with the objective to reduce stigma.

The program combines strategies for education, contact, and skills development. There will be two meetings with the directors of the healthcare centers, and there will be six sessions with health personnel done on a weekly basis for 2 h per session. The program is run by a healthcare professional and a person who has been diagnosed with SMD.

#### Education strategy

Information is presented about stigma and the consequences it has in several areas, especially in health care. The mistaken beliefs about SMD are disproved, specifically topics dealing with danger and violence, and the irreversibility of the disorder. The information is presented by different sources (people with and without a diagnosis of SMD) and through diverse means (orally, audiovisual, and written).

#### Contact strategy

*Direct contact*: There is direct contact throughout the program because the co-facilitator is someone who has a diagnosis of SMD. In one of the sessions, another person with a diagnosis of SMD shares their life testimony.*Indirect contact*: This is initiated through (i) *videos* in which the participants watch and analyze short videos that show different experiences of people with SMD. The videos show situations of stigma and discrimination that those affected have experienced (with health personnel present), as well as good experiences that have happened to them in the context of their health care; (ii) *written cases* that provide information about a person who has received a diagnosis of SMD for the group to discuss; and (iii) *skills strategy* that teaches skills in a practical and contextualized way on how to accept and resolve difficult situations when dealing with people with SMD. This is performed through role play with real cases that have occurred in the healthcare center. Additionally, verbal and non-verbal communication is addressed.

The sessions are designed to encourage group reflection. The information presented will be discussed and analyzed in groups in a way that favors dissonance and cognitive categorization. Furthermore, the program incorporates effective implementation of non-stigmatizing practices through the planning and execution of behaviors that increase positive attitudes and respectful and inclusive treatment toward people who have SMD (47). The program format emphasizes contact conditions that favor its effectiveness, i.e., status equality between the participants, achievement of common goals, and establishment of a cooperative relationship (52). Furthermore, the contact strategies focus on favoring empathy, which is inversely related to prejudice.

### Program Implementation

Meetings will be scheduled with the directors of the healthcare centers as an encouragement to value the intervention and to facilitate and motivate workers to participate. Thus, it is mandatory that at least one of the meetings be prior to the sessions with healthcare workers. The meetings with the directors are conducted with a methodology similar to that used with the workers. Additionally, there will be a facilitator who is a health professional that has undergone a 20 h contact training process, plus several hours of study of written material regarding SMD and the characteristics and effects of stigma. In addition, all the facilitators for the intervention will be empirically trained. Co-facilitators will be people who have been diagnosed with SMD and they will receive 28 direct hours of training. Training times will be different for the facilitators who are health professionals and those who are diagnosed with SMD; however, there are training times that will be shared between the two groups. During the implementation, the facilitators, and co-facilitators will receive weekly supervision, either online or in person, to evaluate the process and correct any difficulties. The facilitators must complete a weekly checklist of activities to ensure that the program is following the established protocol. Additionally, there will be live supervision in which a member of the research team attends the session but does not participate, and then completes a standardized evaluation form on the session. Afterwards, feedback will be given to the facilitator and co-facilitator about their performance.

### Measurements

#### Social Distance Scale (SD)

Social distance scale (SD) Link et al. (73). This scale evaluates the social distance that people have toward people with SMD. It is composed of a brief vignette where the case of a person with SMD is revealed followed by seven items in a Likert-type response with five alternative answers ranging from disagree to completely agree. The questions are related to different situations that vary in the degree of closeness to the affected person who could be a neighbor, friend, employee, or partner. This scale was adapted and validated for use in the Chilean population (74). The final questionnaire is composed of two factors: “closeness and social interaction” (three items) and “intimacy and trust” (two items). The internal consistency of each of the factors reached Cronbach's alpha values of 0.82 for factor 1, 0.75 for factor 2, and 0.78 for the total scale.

CAMI scale (75). This scale evaluates the attitudes of the general public toward people with SMD. The response is a Likert-type format that is based on five alternatives that range from totally agree to totally disagree. The original scale was adapted and validated for use in the Chilean population (76). The scale is made up of two factors, “acceptance” and “rejection of the installation of mental healthcare centers in the community,” each composed of five items. The internal consistency for each of the factors reached Cronbach's alpha values of 0.61 for the first factor, 0.66 for the second factor, and 0.69 for the total scale.

“Mental Illness: Clinicians' Attitudes” (MICA) scale (77). MICA is a 16-item scale that evaluates the attitudes of professionals and students working in health toward people with SMD. The questionnaire consists of six answer options in a Likert format that ranges from strongly agree to strongly disagree. The internal consistency of the original scale was good (α = 0.72). An adaptation of this scale is being made for validation in the Chilean population.

Self-registration of inclusive behaviors: This is an *ad hoc* scale created to assess self-perception of behaviors that one had toward the last person diagnosed with SMD with whom they had contact. There are 18 items with four response options in a Likert format.

### Procedure

Primary healthcare centers from the province of Concepcion, Chile will be invited to participate. The participation of each center is approved by the Ethical-Scientific Committee of each health service and authorized by their directors. A requirement for the inclusion of a center in the study is the authorization for the participation of health workers in the program sessions during work hours. Various activities will be performed to promote the program in each selected health center and to encourage participation. The program will be implemented in health centers where there are at least 18 people enrolled to permit the randomization of control and experimental groups with at least nine people per group. Given the sizes of the centers, a maximum number of 40 participants will be considered per health center since it is unlikely that there will be more than that number of people enrolled. The randomization process will form groups with 9 to 20 people. All healthcare workers will be invited to participate through informative meetings and flyers distributed in each health center. Once the participants have agreed to take part in the program and have been evaluated, they will be randomized. The initial evaluation (T1) will take 3 weeks, and the implementation of the program will last a total of 8 weeks. The post-test evaluation (T2) will be done during the 3 weeks following the end of the program. Four months after the end of the program, the follow-up evaluation will be performed (T3) (Table 1).

**Table 1:** Study assessment points.

**Measurement**	**Scale**	**T1**	**T2**	**T3**
**SOCIODEMOGRAPHIC DATA**				
Sociodemographic data	Sociodemographic characterization file	✔	–	–
**STIGMA IN PRIMARY HEALTHCARE WORKERS TOWARD PEOPLE WITH DIAGNOSIS OF SEVERE MENTAL DISORDERS (SMD)**				
Attitudes of the general public toward people with SMD	Community Attitudes to Mental Illness (CAMI) scale	✔	✔	✔
Attitudes of professionals and students working in health toward people with SMD	Mental Illness: Clinicians' Attitudes (MICA) scale	✔	✔	✔
Social distance that people have toward people with SMD	Social distance scale (SD)	✔	✔	✔
Self-perception of behaviors that one had toward the last person diagnosed with SMD with whom they had contact	Self-registration of inclusive behaviors	✔	✔	✔

*T1, assessment before intervention; T2, assessment after intervention; T3, follow-up 4 months after assessment T2*.

Workers assigned to the control group will receive the program once the implementation is completed and the post and follow-up evaluations are finished. A minimum of 11 centers are needed to obtain the required sample size (Table 1).

### Power and Data Analyses

An experimental multilevel mixed effects design with effects between groups (treatment vs. control) and within groups (time), with pre- and post-test measurements and with a four-month follow-up, will be used. Estimation of the effect will be by intention-to-treat. To estimate the effect of the intervention on post-test measurements, it should be taken into account that there are multiple outcomes and the results per person are not independent of each other but can have distinct distributions. Therefore, a multivariate analysis of covariance (MANCOVA) will be used where the presence of clustering per healthcare center is considered as a factor, while the pre-test measurement and previous contact with someone with SMD are considered as co-variables. Previous verification of the assumptions that the model provides, i.e., presence of normal multivariate distribution of the transformation of the measurements, as well as homogeneity of the variance/covariance matrix between the groups under study, is necessary.

Once it has been determined that the intervention is successful in general terms, specific analyses of each result will be performed using mixed ANCOVA that considers participation in the control group or experimental group as a fixed factor of interest, pre-measurement as a co-variable, and enrollment in the primary healthcare center as a random factor. To detect a moderate effect for the intervention (*d* = 0.5) with a power of 95% and a level of significance of 5%, a minimum sample of 105 participants per group is estimated (210 participants in total). This number was obtained by performing a simulation procedure using a mixed ANOVA considering the existence of a significant interaction effect between moment X group experimental/control. Considering a 10% loss in percentage, 231 is the minimum estimated number of participants.

## Discussion

It is expected that an intervention program that recognizes the aforementioned aspects will reduce the stigmatization that healthcare workers have toward people with SMD. Reducing stigma toward people with SMD should be one of the priorities of a policy aimed at ensuring the rights of people with psychiatric diagnoses and guaranteeing equity in health care. The results of this study could help future research to evaluate the potential of the program to be part of a global strategy geared toward improving equity in health care and the quality of life of people with SMD and their families.

One limitation of this study is the risk of contamination between the control and experimental groups within each healthcare center. However, it was estimated that this risk was lower given the characteristics of the centers and the program, which assumes that attitudinal change is not easily achieved and that direct contact with people having SMD is decisive. This experience cannot be transmitted to other people without a substantial effort. Therefore, considering the working conditions of the healthcare personnel (high demand and overload), group crosstalk is not expected to frequently occur, thereby decreasing the possibility of contamination. Furthermore, the study design of this intervention program avoids the methodological difficulties of clustered designs, which are the preferred choice to have less risk of contamination. The results of this study will be published in scientific journals and scientific meetings, and it will also be presented to the general public, especially to those involved in protecting the rights of people with SMD, and to health authorities. The data of this study will be available to those who request it.

## Ethics Statement

This study was approved by the Scientific Ethics Committee of the corresponding Health Services and was designed taking into consideration the rights of the participants included in the Declaration of Helsinki and the principles of respect, autonomy, and beneficence. All participants (professionals and technicians) were invited to participate, and if they accepted, they were asked to express their agreement by signing an informed consent form. In the case of the members of the organization of people diagnosed with SMD, they are accustomed to participate in anti-stigma activities since it is one of the objectives of the organization. It is well documented that this type of activity improves the esteem and confidence of people with SMD and does not violate their integrity or rights; on the contrary, it favors the process of their recovery (50).

## Author Contributions

PG primary investigator. SS and FC were involved in study design. FC and PV were involved in the selection of measurements. RR-V, VV, CZ, and CO was involved in the design of the intervention.

### Conflict of Interest Statement

The authors declare that the research was conducted in the absence of any commercial or financial relationships that could be construed as a potential conflict of interest.
